# The Highly Virulent 2006 Norwegian EHEC O103:H25 Outbreak Strain Is Related to the 2011 German O104:H4 Outbreak Strain

**DOI:** 10.1371/journal.pone.0031413

**Published:** 2012-03-05

**Authors:** Trine M. L'Abée-Lund, Hannah J. Jørgensen, Kristin O'Sullivan, Jon Bohlin, Goro Ligård, Per Einar Granum, Toril Lindbäck

**Affiliations:** Department of Food Safety and Infection Biology, Norwegian School of Veterinary Science, Oslo, Norway; University of Hyderabad, India

## Abstract

In 2006, a severe foodborne EHEC outbreak occured in Norway. Seventeen cases were recorded and the HUS frequency was 60%. The causative strain, *Esherichia coli* O103:H25, is considered to be particularly virulent. Sequencing of the outbreak strain revealed resemblance to the 2011 German outbreak strain *E. coli* O104:H4, both in genome and Shiga toxin 2-encoding (Stx2) phage sequence. The nucleotide identity between the Stx2 phages from the Norwegian and German outbreak strains was 90%. During the 2006 outbreak, *stx_2_*-positive O103:H25 *E. coli* was isolated from two patients. All the other outbreak associated isolates, including all food isolates, were *stx*-negative, and carried a different phage replacing the Stx2 phage. This phage was of similar size to the Stx2 phage, but had a distinctive early phage region and no *stx* gene. The sequence of the early region of this phage was not retrieved from the bacterial host genome, and the origin of the phage is unknown. The contaminated food most likely contained a mixture of *E. coli* O103:H25 cells with either one of the phages.

## Introduction

Enterohaemorrhagic *Escherichia coli* (EHEC) can cause serious disease in humans. Infection manifests itself as diarrhoea or haemorrhagic colitis. The life threatening haemolytic uraemic syndrome (HUS) is a potential sequelae. Previously, EHEC isolates belonging to serogroups O157, O26, O111, O145 and O103 were most frequently isolated in food borne outbreaks [1]. Recently, less common serotypes and pathotypes, have received more attention. This is illustrated by the enteroaggregative *E. coli* (EAEC) O104:H4 causing a large European outbreak in 2011 involving more than 4000 diseased patients, a 22% HUS incidence, and 50 fatalities [2], [3].

EHEC virulence is mainly attributed to the production of Shiga toxins (Stx), which are regarded as essential in EHEC disease. Shiga toxins are divided into two major families, Stx1 and Stx2. Toxins belonging to the Stx2 group are the most heterogenic and also include the most potent variants [4]. In *E. coli*, Stx are usually encoded by temperate, lamdoid bacteriophages whose genomes are mosaic in structure, and may integrate at several sites in the *E. coli* chromosome [5]–[7]. Upon certain stimuli of the bacterial cell, the phage enters a lytic cycle inducing production of Stx and phage particles. This culminates in bacterial cell lysis and release of toxin and infectious phages [8], [9]. Released Stx phages may infect new bacterial cells, playing an important role in the evolution of EHEC [10].

During the spring of 2006, Norway experienced a national disease outbreak caused by EHEC O103:H25. The outbreak was characterized by an extraordinary high frequency of HUS. Of seventeen recorded cases sixteen had diarrhoea, ten developed HUS and one case was fatal [11]. Stool cultures for *E. coli* O103:H25 were positive in 11 of the patients, but only two of the retrieved isolates were *stx_2_*-positive while the remaining nine were *stx*-negative [11]. The absence of *stx* genes in EHEC serotypes isolated from patients is not uncommon, and such strains are believed to be offspring of EHEC present at an earlier phase of the infection [12]. The more surprising finding in the Norwegian O103:H25 outbreak was that none of the food isolates had *stx*-genes [13]. Genotyping and MLVA revealed that the *stx*-negative isolates from food were indistinguishable or closely related to the two *stx_2_*-positive patient isolates [13], [14], and these results combined with epidemiologic investigation led to the conclusion that fermented sausage with the *stx*-negative isolates was the culprit of the outbreak [11], [13].

In this study, the genome of the outbreak strain was sequenced to elucidate its high virulence and examine potential relationship to other virulent EHEC strains. In addition, we aimed to characterize the Stx2 phage demonstrated in the *stx_2_*-positive isolates.

## Results

### Genome sequencing and phylogeny

**Figure 1:**
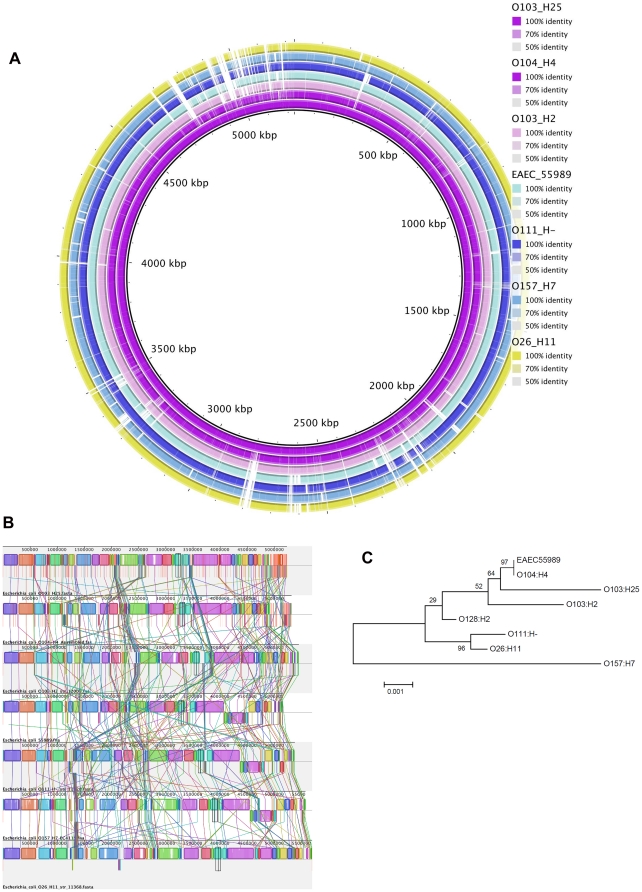
Genome comparisons of EHEC O103:H25 NOS and related *E. coli* strains. A. BRIG blast atlas of EHEC O103:H25 NOS compared to EAEC O104:H4 GOS, EHEC O103:H2 str 12009, EAEC O104:H4 str 55989, EHEC O111:H- str 11128, EHEC O157:H7 EDL933 and EHEC O26:H11 str 11368. The white regions represent absent genetic regions. B. Whole genome alignment of EHEC O103:H25 NOS, EAEC O104:H4 GOS1, EHEC O103:H2 str 12009, EAEC O104:H4 str 55989, EHEC O111:H- str 11128, EHEC O157:H7 EDL933 and EHEC O26:H11 str 11368 (top to bottom) genomes using Mauve [15]. Each chromosome has been laid out horizontally and homologous blocks in each genome are shown as identically colored regions linked across genomes. C. Phylogenetic analysis of concatenated MLST gene alleles (*adk*, *fumC*, *gyrB*, *icd*, *mdh*, *purA*, *recA*) of EHEC O103:H25 NOS (ST2523), EAEC O104:H4 GOS (ST678), EAEC O104:H4 str 55989 (ST678), EHEC O103:H2 str 12009 (ST17), EHEC O26:H11 str 11368 (ST21), EHEC O111:H- str 11128 (ST16), EHEC O157:H7 EDL933 (ST11) and EHEC O128:H2 str 3171/00 (ST25) obtained from GenBank.

The sequence achieved through genome sequencing using 454 technology was assembled into 554 contigs and spanned altogether 5.2 Mb. Figure 1A shows a BRIG diagram comparing the EHEC O103:H25 NOS genome against the *E. coli* genomes of O104:H4 GOS1, O103:H2 str 12009, O104:H4 str 55989, O111:H- str 11128, O157:H7 and O26:H11 str 11368, revealing that EHEC O103:H25 NOS shows considerable resemblance to the German outbreak strain, EAEC O104:H4 GOS1.The Mauve alignment [15] shown in Figure 1B supports this finding. EHEC O103:H25 NOS was assigned to the sequence type 2523 (ST2523) complex (*adk* 77, *fumC* 7, *gyrB* 7, *icd* 151, *mdh* 65, *purA* 56, *recA* 7). A phylogenetic tree of allele sequences supports a close relationship between EHEC O103:H25 NOS and EAEC O104:H4 GOS (ST678 [16]) (Figure 1C).

### Virulence features

All five LEE operons (LEE1 to LEE5) comprising the LEE core of 35 kb [17] were present in the EHEC O103:H25 NOS. The intimin gene, *eae* θ (theta), was present in LEE5. The sequence of the five LEE operons showed 97% and 86% identity to EHEC 0111:H- str 11128 and EHEC O103:H2 str 12009, respectively.

A complete OI122, including the genes *sen* (Shigella flexneri enterotoxin 2), *pagC* (required for bacterial survival within macrophages) and *efa* (EHEC factor for adherence – an adhesin) [18], was present in all of the Norwegian O103:H25 isolates listed in Table 1. The OI122 in these isolates exhibits the large version of *efa/lifA* gene, an orf of 9672 bp, which encodes a protein inhibiting lymphocyte activation and lymphokine production [19]. The genes encoding enterohemolysin (*ehxCABD*) were all present in the O103:H25 NOS genome, and *ehxA* was detected in all Norwegian strains listed in Table 1. Neither this subtilase cytotoxin genes *subA-subB*, nor the genes *pic*, *aggA*, *aggR*, *aatA* associated with enteroaggregative *E. coli*, was present in EHEC O103:H25 NOS or any of the other outbreak isolates included in this study.

**Table 1:** Bacterial isolates included in the study.

Strain	Synonym	Year	Stx	Source	Origin
NVH-734^a^	NIPH-11060424	2006	*stx_2_*	Patient no 2	Norway
NVH-847	NIPH-11060708	2006	*stx_2_*	Patient no 9	Norway
NVH-848	NIPH-11060707	2006	-	Patient no 9	Norway
NVH-849	NIPH-11060747	2006	-	Patient no 10	Norway
NVH-760	625/06	2006	-	Fermented sausage, home of patient 7	Norway
NVH-737		2006	*-*	Fermented sausage	Norway
NVH-763		2006	*-*	Food	Norway
NVH-661	NIPH-10306923	2003	*stx_2_*	Patient	Norway
NVH-731	NIPH-11051601	2005	*stx_2_*	Patient	Norway
cdc-08-201				Not known	Maine, USA
cdc-08-202				Not known	Virginia, USA

All isolates are *E. coli* O103:H25.

a Referred to as Norwegian outbreak strain (NOS).

Characteristics and virulence features of EHEC O103:H25 NOS compared to one of the first described EHEC isolates O157:H7 EDL933 and the related strains EAEC O104:H4 GOS [3], [16] and EHEC O103:H2 str 12009 are listed in Table 2.

**Table 2:** Characteristics of EHEC O103:H25 NOS, the related strains O104:H4 GOS and O103:H2 str 12009, and the EHEC reference strain O157:H7 EDL933.

Characteristics	O157:H7 EDL933	O103:H25NOS	O104:H4 GOS	O103:H2str 12009
**Outbreak**				
Year of isolation	1982	2006	2011	2001
Country	USA	Norway	Germany	Japan
Pathotype	EHEC	EHEC	EAEC	EHEC
No of diseased	47	16	4075	1
No of HUS (%)	0	10 (62.5%)	908 (22%)	0
No of deaths		1	50	0
**Stx2 phage**				
Insertion site	*wrbA*	*wrbA*	*wrbA*	*argW*
Stx2A, nucleotide position 867	T	C	C	T
**LEE**				
LEE operons	five	five	none	five
Intimin type	gamma	theta	not present	epsilon
**OI122**				
*sen (Shet2)*	yes	yes	no	yes
*pagC*	yes	yes	no	yes
*efa1/lifA*	2.1 kb	9.7 kb	not present	9.7 kb
**Accessory virulence**				
*set1A (shet1)*	no	no	yes	no
Colicin		E2		
*ehxA*	yes	yes	not present	yes

### PFGE analysis

**Figure 2:**
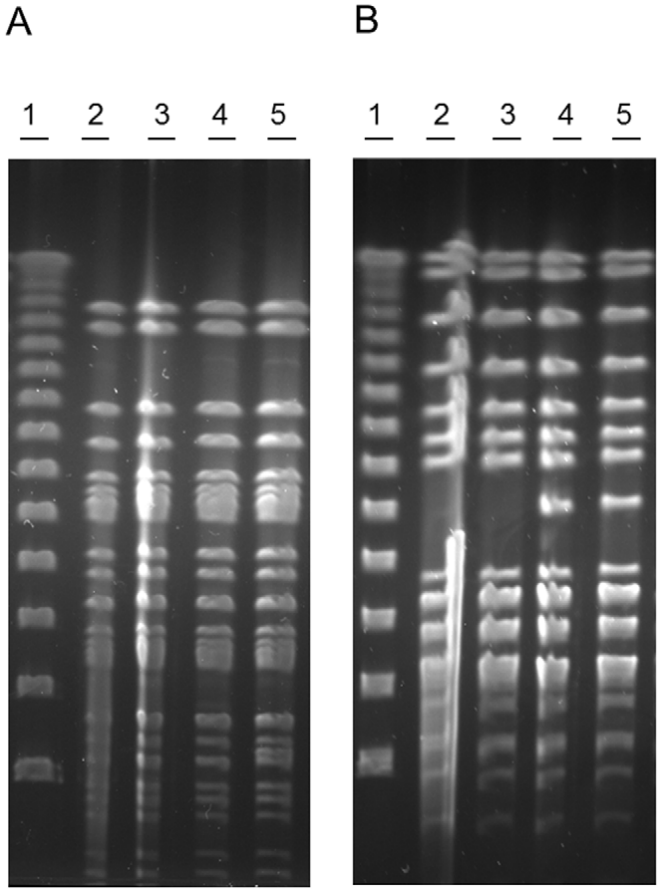
PFGE of outbreak associated isolates. PFGE of *E. coli* O103:H25 NOS (lane 2), NVH-847 (lane 3), NVH-848 (lane 4), and NVH-760 (lane 5). Lambda ladder is used as marker (lane 1). Digestion with *Xba*I (A) showed indistinguishable PFGE patterns, while digestion with *Avr*II (B) exposed a difference between the *stx_2_*-positive and the *stx*-negative isolates.

To distinguish between the *stx_2_*-positive and the *stx*-negative isolates the two *stx_2_*-positive isolates from patients and two *stx*-negative isolates, one from a patient and one from food (isolate NVH-848 and NVH-760, respectively), were analyzed by PFGE using the restriction enzyme *Avr*II. The PFGE patterns from *Xba*I digestion were indistinguishable as previously described by Sekse et al. [13] (Figure 2A), whereas the *Avr*II digestion exposed a difference in the PFGE pattern between the *stx_2_*-positive isolates and the *stx*-negative isolates (Figure 2B).

### Stx2 phage

**Figure 3:**
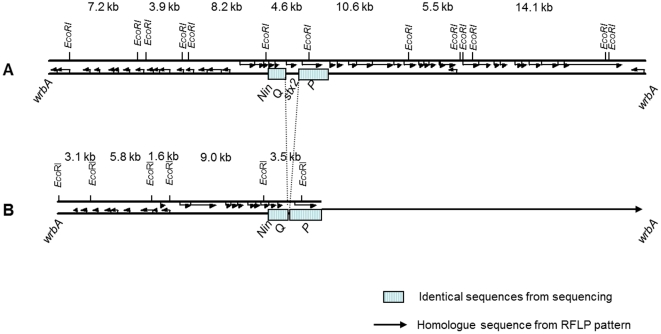
Comparison of Stx2 phage and *stx*-negative phage. Comparison of Stx2 phage and *stx*-negative phage from EHEC O103:H25 NOS (A) and *E. coli* O103:H25 NVH-848 (B), respectively. The Stx2 phage is sequenced, while the illustration of the *stx*-negative phage is based on sequence (23 kb) and RFLP pattern (illustrated by arrow).

**Figure 4:**
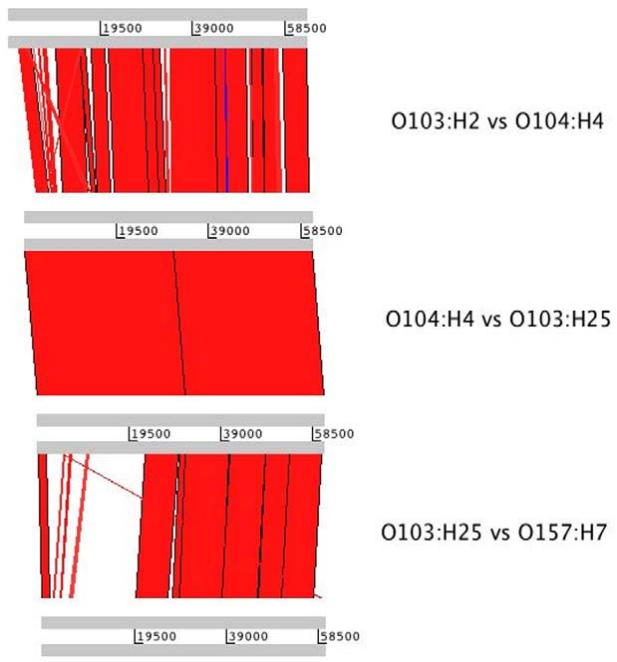
Stx2 phage from EHEC O103:H25 NOS compared to Stx2 phages from EAEC O104:H4, EHEC O103:H2 and EHEC O157:H7. ACT visualization between *stx_2_*-positive phages from EHEC O103:H25 NOS, EAEC O104:H4 str C227-11, EHEC O103:H2 str 12009 and reference Stx2 phage 933W from EHEC O157:H7 EDL933. The phage genomes are compared using BLAST and the red regions represent hits. The white regions indicate absent genetic regions, which is especially noticeable in the comparison between the O103:H2 phage and the O157:H7 phage.

To retrieve the genome sequence of the Stx2 phage, contigs from the EHEC O103:H25 NOS genome sequence containing Stx2 phage DNA were assembled, followed by gap closure PCRs and sequencing. The Stx2 phage genome is 60595 bp in length with a G+C content of 50%. A schematic view of the Stx2 phage is shown in Figure 3. The entire phage sequence was blasted against the sequence of the Stx2 phages of EAEC O104:H4 str C227-11, EHEC O103:H2 str 12009 and EHEC O157:H7 EDL933, and an ACT comparison is shown in Figure 4. The nucleotide identity to the Stx2 phages of EAEC O104:H4 str C227-11 [2] and EHEC O103:H2 str 12009 [7] is 90 and 88%, respectively, and covers the entire sequence, while the similarity to the reference EHEC O157:H7 EDL933 phage is limited to the 35 kb late region (Figure 4).

### Stx2-related phage

**Figure 5:**
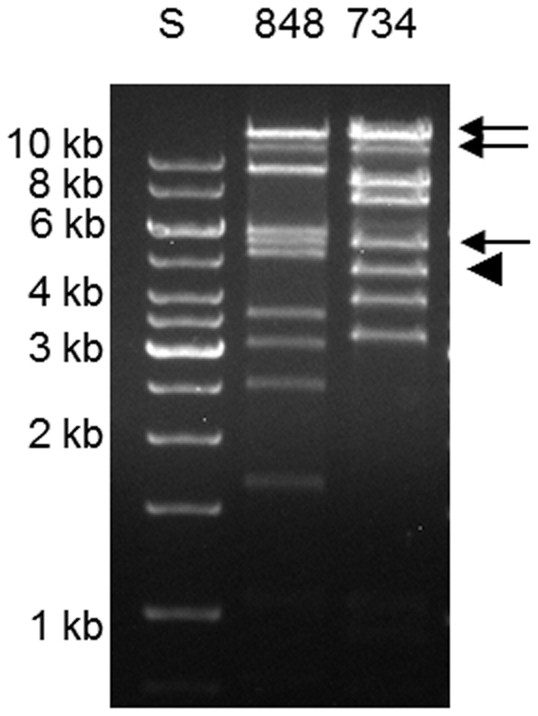
RFLP of phage from *stx_2_*-positive and *stx*-negative isolates. *Eco*RI restriction fragment length polymorphism analysis of Stx2 phage and *stx*-negative phage in EHEC O103:H25 NOS and *E. coli* O103:H25 NVH-848, respectively. The arrow indicates the *Eco*RI fragments that are indistinguishable between the strains, arrowhead indicates the *Eco*RI fragment of *E. coli* O103:H25 NOS where the *stx_2_* gene is located.

In plaque hybridization assays, all plaques produced by EHEC O103:H25 NOS were *stx_2_* positive, while all plaques produced by *E. coli* O103:H25 NVH-848 were *stx_2_* negative. DNA from these latter phages (hereafter referred to as *stx*-negative phage) was isolated via plaque assay. RFLP patterns showed that phages from five *stx*-negative isolates were identical, but the restriction pattern differed from that of the phages of the two *stx_2_*-positive patient isolates (Figure 5). The integration site for the *stx*-negative phage was *wrbA*, which is the same site as the Stx2 phage [26].

Hybridization located *stx_2_* to a 4.5 kb *Eco*RI fragment not present in the *stx*-negative phage RFLP (Figure 5). Still, RFLP analysis revealed three bands of about 14, 11 and 5.5 kb that were common between the *stx_2_*-positive and -negative phages (Figure 5). The estimated sizes of the three bands correspond to the three large *Eco*RI fragments comprising the late region of the sequenced Stx2 phage NOS (Figure 3), indicating that the late region of the two phages are homologue sequences.

**Figure 6:**
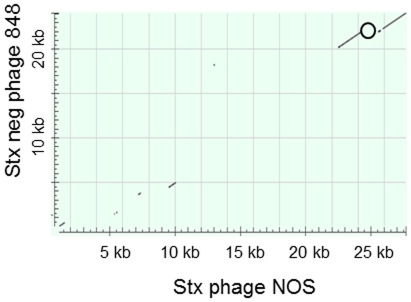
Early region of Stx2 phage compared to early region of *stx*-negative phage. Dot matrix view of 24 kb early region of the Stx2 phage from EHEC O103:H25 NOS and the 23 kb early region of the *stx*-negative phage from *E. coli* O103:H25 isolate NVH-848. The position of the *stx_2_* gene in EHEC O103:H25 NOS is marked (o). Regions of similarity are based upon the BLAST results. Alignments are shown in the plot as lines. Plus strand and protein matches are slanted from the bottom left to the upper right corner, minus strand matches are slanted from the upper left to the lower right. The number of lines shown in the plot is the same as the number of alignments found by BLAST.

To sequence the early region of the *stx*-negative phage, two fragments of 3.2 and 1.6 kb (Figure 5) were cloned and sequenced. Primer walking was used to amplify PCR products from the *stx*-negative phage DNA to complete the sequence. The 23 kb sequence of the early region of the *stx*-negative phage is not related to the Stx2 phage from O103:H25 NOS and a schematic view of the two phages is shown in Figure 3. The sequence of the *stx*-negative phage revealed an *Avr*II restriction site not present in the Stx2 phage genome. The early phage regions of the two phages were blasted against each other and a Dot matrix view is shown in Figure 6.

Contigs from the EHEC O103:H25 NOS genome did not contain the sequence of the *stx*-negative phage, and also PCR run on total DNA from EHEC O103:H25 NOS with primers specific for the early region of the *stx*-negative phage were negative or gave products of incorrect size.

### Cloning of phage DNA

An attempt to isolate and clone phage DNA directly from EHEC O103:H25 NOS resulted in the discovery of another phage in this strain. This phage is 45 kb, has a G+C content of 47% and the sequence is 53% identical to bacteriophage ΦV10 which is a temperate phage that specifically infects *E. coli* serogroup O157:H7 [20]. The insertion site of the phage is within the *guaA* gene, and the phage does not infect *E. coli* DH5α.

### Colicin and plasmids

Colicin from EHEC O103:H25 NOS in culture supernatants was phenotypically observed as clear zones in an *E. coli* DH5α cell lawn. *In silico* analysis of the sequenced genome demonstrated the presence of a colicin E2 gene and its associated immunity and lysis genes on a 6744 bp contig. A gap-closure PCR confirmed the contig to be a complete plasmid. Hybridization with a *colE2* pcr template identified the presence of the colicin E2 carrying plasmid in outbreak-associated O103:H25 isolates from both patients and food, including the isolates from 2003 and 2005, while non-outbreak O103:H25 isolates associated with Norwegian sheep did not harbour the plasmid. The entire colicin plasmid was 97% identical to pO111_4 from *E. coli* O111:H- str 11128 [7].

**Figure 7:**
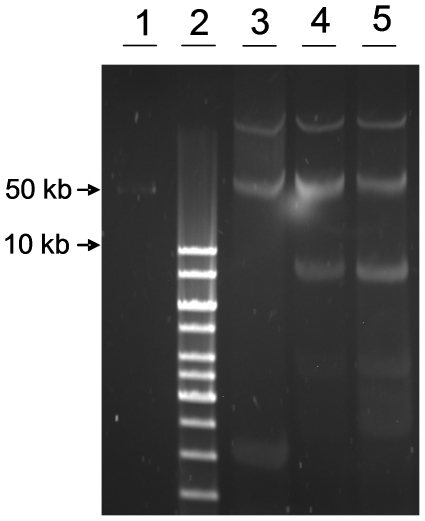
Plasmid profiles of *E. coli* O103:H25. Comparison of large plasmids in EHEC O103:H25 NOS (lane 4) and *E. coli* O103:H25 NVH-848 (lane 5) with EHEC O157:H7 EDL 933 (lane 3). GeneRuler 1 kb ladder (lane 2) and Lambda ladder (lane 1) were used as molecular size marker.

EHEC O103:H25 NOS and *E. coli* O103:H25 NVH-848 share identical plasmid profiles and harbour one large plasmid of approximately the same size as pO157 of *E. coli* O157:H7 EDL 933 (Figure 7).

## Discussion

Serotype O103:H25 is a rare cause of EHEC disease, and has not previously been associated with outbreaks [11], only with sporadic disease cases [21], [22]. A few *E. coli* O103:H25 strains have been characterized and found to carry *stx_1_*, but they have not been associated with severe disease [18]. The Norwegian 2006 outbreak caused by EHEC O103:H25 had a 60% HUS frequency, and the strain is considered to be particularly virulent [11].

We have shown that the genome of EHEC O103:H25 NOS resemble the EAEC O104:H4 GOS and EHEC O103:H2 str 12009. This is supported in a pan genomic study of 61 sequenced *E. coli* genomes, showing that the *E. coli* O103 Oslo (O103:H25 NOS) clusters closely together with the EHEC O103:H2 str 12009 [23], and a more recent study shows the similarities between the EHEC O103:H2 str 12009 and the EAEC O104:H4 GOS genomes [2]. EAEC O104:H4 GOS caused diarrhoea in approximately 4000 individuals, 22% of which developed HUS, and the strain is notably more virulent than most EHEC [2]. The *E. coli* O104:H4 outbreak began in Germany in May 2011, but was later identified in other European countries [2], [24]. Due to phenotypic and genotypic characteristics, the German O104:H4 outbreak strain is not classified as an EHEC, but rather as a Shiga toxin producing enteroaggregative *E. coli* (EAEC) [2], [3], [25]. Despite the lack of genes characteristic of EAEC in EHEC O103:H25 NOS, and thus differing in both pathotype and serotype, the genomes of the Norwegian and the German outbreak strains are highly similar, as illustrated in Figures 1A and 1B. The close relationship between the two strains is supported by MLST analysis (Figure 1C). Also the Stx2 phages in these two strains show a striking homology with a DNA sequence identity of 90% (Figure 4) [2]. The identity includes a 1 bp silent nucleotide mutation in the *stx2A* gene [3], [26] which is rare in other *stx_2_* genes. This indicates a common origin for the two phages. The finding of closely related Stx2 phages and genomes in two outbreak strains of different serotypes and pathotypes, but with a high HUS incidence in common, is remarkable and will be investigated further.

Other strains with related Stx2 phages include EHEC O103:H2 str 12009 from a sporadic case of diarrhoea in Japan in 2001, and *E. coli* O111:H- str 11128. Similar Stx2 phages to the Norwegian outbreak strain are thus present in *E. coli* strains of serogroups O103, O104 and O111, and the phage seems to be rather promiscuous in nature. The similarity between the Stx2 phage of EHEC O103:H25 NOS and the reference Stx2 phage 933W from O157:H7 EDL933 is high in the 38 kb late region of the phages where the *stx* genes are located (95%), however, the early regions of these phages differ in composition (Figure 4). In EHEC O103:H25 NOS the Stx2 phage is inserted into *wrbA*, a previously described integration site of Stx2 phages, e.g. in EHEC O157:H7 EDL933 and Sakai strains [27], [28]. The *wrbA* had not been observed as an integration site in serogroup O103 prior to the Norwegian outbreak [26]. The closely related Stx2 phage in O104:H4 GOS is also inserted in *wrbA*, while the Stx2 phage in EHEC O103:H2 str 12009 is located within the *argW* gene.

The only observed feature distinguishing between the Norwegian outbreak strain and *stx*-negative isolates from the 2006 outbreak is the presence of either the *stx_2_*-positive or the *stx*-negative phage, respectively. These two phages are related and share parts of their sequences and insertion site. While the 30 kb late regions are similar in the two phages, the early regions are completely different. Interestingly, the shift between the similar and dissimilar parts is abrupt and in proximity of the *stx* genes (Figure 3). As Stx2 phages are mosaic by nature and rearrangements are not uncommon [6], the *stx*-negative phage could have developed from the Stx2 phage by acquiring its distinctive sequence from the chromosome of the *E. coli* O103:H25 host. However, the distinctive *stx*-negative phage sequence has not been identified in the EHEC O103:H25 NOS genome neither by *in silico* analysis nor by PCR. This indicates that this phage, or at least part of it, has another origin. The distinctive sequence of the *stx*-negative phage shows some similarities to phage related sequences in a BLAST search, but it seems to have a rather unique construction.

The 2006 EHEC O103:H25 outbreak is remarkable because all food isolates were *stx*-negative, and only two of 11 isolates from patients were *stx_2_*-postive [11], [13]. The lack of *stx* genes in EHEC serotypes isolated from patients is not uncommon and has been reported in both O157 and non-O157 isolates [12], [29]. In studies where the mechanism of *stx* gene loss has been investigated, it has been shown that the *stx*-negative strains lack the entire Stx-encoding bacteriophage. This has been demonstrated by the presence or reappearance of an intact integration site for the Stx phage and an altered PFGE pattern [29]–[32]. Such *stx*-negative isolates from patients are believed to be progenies of an EHEC that lost the *stx* genes during the course of illness, and might be referred to as EHEC-LST (lost Shiga toxin) [12]. The EHEC-LST model is supported by the finding that EHEC are difficult to isolate from patients late in illness [33], and the theory is further confirmed by Mellmann and Karch who demonstrated the presence of *stx*-negative strains of O26:H11/NM and sorbitol fermenting (SF) O157:NM subsequent to *stx_2_*-positive isogenic isolates in the same patients [12], [34], [35].

In contrast to the EHEC-LST phenomenon, we find that the integration site of the Stx2 phage is occupied in the *stx*-negative isolates by a partly related phage but without *stx* genes. The *stx*-negative isolates thus differ from the *stx_2_*-positive isolates not only by the lack of the Stx2 phage but also by the presence of this *stx*-negative phage. The similar size and the lack of an *Xba*I restriction site in both the Stx2 phage and the *stx*-negative phage explain the finding of identical *Xba*I digested PFGE profiles of the *stx_2_*-positive and *stx*-negative isolates shown by Sekse et al. [13]. Digestion with *Avr*II, however, revealed a difference in PFGE pattern, and sequencing of the two phage genomes identified an *Avr*II restriction site in the Stx2 phage which is not present in the *stx*-negative phage and that most likely explains the observed difference.

Only two *stx_2_*-positive patient isolates were retrieved in 2006, and as no *stx_2_*-positive isolates were retrieved from food, it could be speculated that the *stx*-negative *E. coli* acquired the Stx2 phage in the patients' gut. However, the Stx2 phages from the two patient isolates have identical RFLP pattern and a rare silent nucleotide mutation in *stxA*, which both are found in two EHEC O103:H25 isolates from sporadic cases in Norway in 2003 and 2005 [26]. This strongly indicates that the Stx2 phages in these four isolates are epidemiologically linked and that the *stx_2_*-positive isolates from 2006 originates from the same source.

The peculiar circumstance is that both *stx_2_*-positive and *stx*-negative O103:H25 *E. coli* cells must have been present in the contaminated fermented sausage in 2006. Which of the two variants is the ancestor is difficult to predict, but there is reason to believe that the *stx_2_*-positive clone preceded the *stx*-negative clone because the related Stx2 phage was identified in the same *E. coli* serotype three years prior to the 2006 outbreak. On the other hand, *stx*-negative isolates could have been overlooked in earlier cases, and the origin and history of this clone is difficult to evaluate. The hypothesis that bacterial cells with *stx*-negative or *stx_2_*-positive phage were both present in the food is strengthened by the finding of Sekse et al. [26]. They were not able to isolate infective phage particles from food samples in the 2006 outbreak, but *stx_2_* was detected in food samples by PCR. Although the food most likely contained a mixture of *stx_2_*-positive and *stx*-negative bacterial cells, the two clones may not have been present in equal numbers. As only the *stx*-negative clone is isolated from food the proportion of *stx_2_*-positive *E. coli* was probably considerably lower. In the diseased patients, this imbalance might have been reversed during the course of illness. All investigated non-*stx_2_* isolates from patients have been shown to be the same *stx*-negative clone as is isolated from food, and not offspring from the *stx_2_*-positive clone in form of EHEC-LST.

Several factors may contribute to the virulence of EHEC including factors within the individual (age, microbiota, number of Gb3 receptors), the bacterial host (intimin type), the toxin itself (subtype, synergy, Stx toxin levels), and other virulence factors (e.g. subtilase cytotoxin) [4], [18], [36]–[38]. The pathogenicity island locus of enterocyte effacement (LEE) is associated with virulence, and encodes the intimin gene (*eae*), the translocated intimin receptor (Tir) and a type III secretion system (TTSS). These are all involved in the intimate attachment of EHEC to enterocytes [39]. All five LEE operons are present in EHEC O103:H25 NOS, however, LEE is not part of the EAEC O104:H4 GOS genome. Another genomic island present in the 2006 outbreak strain is the putative pathogenicity island OI122 with the genes *sen*, *pagC* and *efa1* which have been strongly correlated with virulence and disease severity [18], [40]. The complete *efa1*/*lifA* gene is 9672 kb and is a bifunctional protein for adherence and inhibition of lymphocyte activation [19]. The distribution of this large toxin is limited to less than 30 strains of heterologous serogroups (BLAST search), only one of the sequenced O157:H7 strains and two O157:NM strains exhibit it [41], [42]. EAEC O104:H4 GOS does not exhibit any version of *efa/lifA*, while O103:H2 str 12009 exhibits the large version of *efa/lifA*. Enterohemolysin (Ehx) is regarded a virulence factor of EHEC and the genes are located on large plasmids like pO157 [43]–[45]. One large plasmid is detected in O103:H25 NOS, in contrast to O104:H4 GOS which carries two large plasmids [25].

The colicin production of *E. coli* O103:H25 may have provided an advantage for the bacterial cells in the harsh competition of the gut, making the colonization more efficient. In addition, the colicin E2 is a DNAse colicin which has been found to increase the *in vitro* production of Stx in EHEC cells exposed to it [46]. It is however unlikely that colicin E2 can affect the production of Stx when the genes coexist in the same cell, but it has been shown that intestinal *E. coli* cells can act as chaperones and contribute to the production of Stx [47] and the colicin could possibly play a role here. Similar colicin plasmids are found in EHEC O26:H11 and EHEC O111:H- [7], and in a study by Karama et al. [48], 38.4% of *E. coli* O103:H2 strains were found to have a colicin producing phenotype.

The phi-like phage isolated from the wild-type EHEC O103:H25 NOS has an unknown function in the *E. coli* O103:H25 host. However, the number of the phi-like phage particles released from EHEC O103:H25 NOS after induction with Mitomycin C is estimated to be approximately ten times the number of Stx2 phage particles released (data not shown), and hence this abundant phi-like phage is a practical challenge as it complicates the isolation of the Stx2 phage and *stx*-negative phage directly from wild types.

### Conclusion

Both the Stx2 phage and the bacterial genome from EHEC O103:H25 NOS are related to the Shiga toxin producing EAEC O104:H4 that caused a large European *E. coli* outbreak in 2011. Two patient isolates from the Norwegian O103:H25 outbreak carry an Stx2 phage, while other outbreak associated isolates carry a related phage in the same insertion site. The two variants, *E. coli* O103:H25 with the Stx2 phage or the *stx*-negative phage, have probably both been present in the contaminated food which caused the Norwegian outbreak.

## Materials and Methods

### Bacterial isolates

The *E. coli* O103:H25 isolates included in the study are presented in Table 1. NVH-734 is the outbreak reference strain and is also referred to as the Norwegian outbreak strain (NOS). *E. coli* DH5α was used as recipient strain in the plaque assay. *E. coli* strains were cultured in Luria-Bertani (LB) broth or on LB agar plates (LB containing 1% agar).

### Whole genome sequencing, alignments and Multilocus sequence typing (MLST)

Isolate NVH-734 (EHEC O103:H25 NOS) was sequenced using 454 technology (454 Life Sciences, Branford, Connecticut, USA). Initial genome analysis revealed similarity to the *E.coli* O103:H2 str 12009 (accession: AP010958.1), and this strain was used as template for the alignment of contigs from the EHEC O103:H25 Norwegian Outbreak Strain (NOS) using Mauve [15]. Subsequently, genome alignment of EHEC O103:H25 NOS and the EAEC O104:H4 str German Outbreak Strain (GOS)1 (distributed on 208 contigs, accessions: AFWO01000001.1-AFWO01000208.1) was also carried out with Mauve, using the progressive alignment option. Whole genome BLAST-comparison was performed using the BRIG software package [49] on the following genomes: EHEC O103:H25 NOS, EAEC O104:H4 GOS1, EHEC O103:H2 str 12009, EAEC O104:H4 str 55989, EHEC O111:H- str 11128, EHEC O157:H7 EDL933 and EHEC O26:H11 str 11368. MLST was performed according to Wirth et al. [50], using the seven housekeeping genes *adk*, *fumC*, *gyrB*, *icd*, *mdh*, *purA*, and *recA*. A maximum likelihood test using PhyML [51] was carried out to assess the best nucleotide substitution matrix in R (http://www.R-project.org/) with the package ‘ape’ [52]. Based on this, Tamura-Nei with invariant sites was shown to be the best model, and subsequently MEGA 5 [53] was used to generate a maximum likelihood tree, which was bootstrapped 500 times. Sequence type (ST) of EHEC O103:H25 NOS was obtained from MLST Databases at the ERI, University College Cork (http://mlst.ucc.ie). The O103:H25 NOS, O104:H4 str C227-11 (originating from the German outbreak), O103:H2 str 12009 and O157:H7 EDL933 phages were BLASTed against each other and compared using the ACT tool [54].

### PCR and sequencing

Primers for gap-closure PCR for completing the sequence of the Stx2 phage, and for detection of genes in other isolates than EHEC O103:H25 NOS were designed on the basis of the genome sequence. All PCRs were carried out in an Eppendorf Mastercycler gradient (Eppendorf AG, Hamburg, Germany). DyNAzyme II DNA polymerase (supplied with 10× buffer) and dNTP Mix from Finnzymes (Vantaa, Finland) were used as instructed by the manufacturer. The standard program was as follows: 95°C for 1 min, 30 cycles of 95°C for 1 min, 52°C for 1 min and 72°C for 1 min, and finally 72°C for 5 min. Sequencing of PCR products was performed by Source BioScience geneservice (United Kingdom), and DNA sequences were analyzed using Vector NTI Advance 11 (Invitrogen, Carlsbad, USA) and BLAST. Primers used for PCR detection of other virulence genes are listed in Table 3.

**Table 3:** Primers used in the study.

Primer	Sequence (5′-3′)	Reference	Probe
***colE2***	F ATGAGCGGTGGCGATGGACGC	This study	Hybridisation of plasmid profiles
	R GCCCGGCCATTTGCCACATTCT		
***pagC***	F ATGAGTGGTTCAAGACTGG	[18]	
	R CCAACTCCAACAGTAAATCC		
***sen***	F GGATGGAACCATACCTGG	[18]	
	R CGCAATCAATTGCTAATGC		
***efa1***	F CTCCCAGAGATAATTTTGAGG	[18]	
	R CAACTGTATGCGAATAGTACTC		
***efa2***	F CTGTCAGACGATGACATTGG	[18]	
	R GAAGGATGGGCATTGTGTC		
***stx_2_***	F GCGTTTTGACCATCTTCGT R ACAGGAGCAGTTTCAGACAG	[57]	Hybridisation of RFLP and PFGE

### Plaque assay

*E. coli* O103:H25 LB broth cultures were incubated at 37°C with shaking at 200 rpm to an OD_600_ of 0.3–0.5 (∼2 h). To induce phage production Mitomycin C (Sigma-Aldrich, St. Louis, MO, USA) was added to a final concentration of 0.5 µg/ml and incubation was continued overnight in the dark. The cultures were centrifuged (2000×*g*, 10 min) to remove bacterial cells and debris, and the supernatants (phage lysates) were sterile filtered (0.22 µm; Minisart, Sartorius Stedim Biotech). As the wildtype strains also produced colicin (see below) that lysed the DH5α cell lawn, lysates were treated with 100 µg/µl trypsin (Sigma) at 37°C for 60 minutes prior to use to destroy colicin. The *E. coli* DH5α recipient was grown in LB broth to OD 0.4–0.6 at 37°C and shaking at 200 rpm. For determination of infectious phage particles, 100 µl of tenfold dilutions of phage lysates were added 900 µl *E. coli* DH5α. CaCl_2_ was added to a final concentration of 10 mM. The phage-recipient mixture was incubated for 30 min at 37°C before 2.5 ml molten soft agar (0.7% LB broth) was added and the mixture poured onto LB agar plates with CaCl_2_ (10 mM). The plates were incubated at 37°C overnight, plaques were counted by visual examination and phage titres were calculated.

### Isolation and RFLP analysis of phage DNA

To ensure that DNA was isolated from only the Stx2- or Stx-related phage, DNA extraction was performed via the plaque assay. This was necessary because an abundant phi like phage is also present in the wild type strains, but this phage is not able to infect *E. coli* DH5α. Phage isolation was done from seven strains from the 2006 outbreak, in which five were *stx*-negative strains from either food or patients, and two were *stx_2_*-positive isolates from patients. The isolates are listed in Table 1. Briefly, 0.1 ml from an overnight starter culture of *E. coli* DH5α was transferred to 10 ml LB- broth and incubated at 37°C with shaking until bacterial growth reached mid log phase. Approximately 50 plaque were picked (from the plaque assay described above), dissolved in 50 µl MQ, and added to 0.5 ml of the *E. coli* DH5α culture together with 12.5 µl 1 M CaCl_2_ to facilitate bacteriophage infection. The culture was incubated at 37°C for two hours before 9.5 ml of LB-broth was added and then incubated at 37°C overnight. The overnight culture was centrifuged at 2000×*g* for 10 minutes and the supernatant containing phage particles was sterile filtered (0.22 µm, Minisart, Sartorius Stedim Biotech, Aubagne, France), precipitated with 0.18×PEG 8000/NaCl at 4°C for 2 hours, and centrifuged at 10 000×*g* for 1 hours. The pellet was dissolved in 0.5 ml TE buffer with proteinase K (Sigma-Aldrich, 50 mg/ml) and SDS (final concentration of 0.5%) and incubated for one hour at 56°C. The DNA was extracted using phenol/chloroform/isoamylalcohol (25∶24∶1), and the DNA was precipitated using equal amounts of isopropanol. Phage DNA was used as template in restriction fragment length polymorphism (RFLP) analysis and in PCR reactions. Phage DNA was digested with restriction enzymes *Eco*RI (New England BioLabs, Hertfordshire, England) and the restriction fragments separated by 1% agarose gels. A probe for detection of *stx2A* in RFLP hybridization was made using primers listed in Table 3. To complete the phage genomes of the *stx_2_*-positive and *stx*-negative phages, phage DNA from O103:H25 NOS and NVH-848, respectively, were used as templates in PCR reactions.

### Colicin production

Colicin production was detected by observation of a lytic effect of sterile filtered supernatant from overnight culture of EHEC O103:H25 NOS on *E. coli* DH5 cell lawns. The identification of a colicin E2 encoding gene was performed by *in silico* analysis of the genome sequence, and gap closure PCR was performed to confirm a plasmid configuration of the contig. Primers *colE2*F and *colE2*R (Table 3) and standard PCR conditions were used to generate a probe for plasmid hybridization.

### Pulsed-Field Gel Electrophoresis (PFGE) and plasmid isolation

The *E. coli* isolates associated with the outbreak were analyzed by PFGE as described previously [13] using the restriction enzymes *Xba*I and *Avr*II (New England BioLabs). DNA for detection of colicin E2 encoding plasmids were isolated by Qiagen plasmid purification kit (Qiagen, Hilden, Germany), and separated by electrophoresis in 2% agarose gels. Large plasmids were isolated by the method of Kado and Liu [55] with modifications. Bacteria were grown in 5 ml LB broth (Oxoid) over night at 37°C, 250 rpm of which 1.5 ml were centrifuged. The pellet was suspended in 200 ml cold (4°C) TAE-buffer (40 mM Tris-acetate, 2 mM EDTA, pH 7.6) and 400 µl 50 mM Tris/3% SDS, pH 12.55 were added prior to incubation at 55°C for 60 min. Plasmid DNA was extracted twice with phenol-chloroform (1∶1, vol/vol) and 25 µl were applied directly to 0.7% agarose gel. Plasmids were separated by electrophoresis at 120 V for 3 h at 4°C.

### Southern blotting and hybridization

Plasmid DNA and digested phage DNA were transferred to nylon membranes (Hybond-N, Amersham International plc, Amersham, United Kingdom) by Southern blotting [56]. For detection of the colicin E2 encoding gene and phage genes probes were labelled with digoxigenin (DIG) and hybridized with a DIG DNA labelling and detection kit (Roche Diagnostics, Basel, Switzerland) according to the instructions by the manufacturer.

### Cloning of phage DNA

Phage DNA isolated directly from EHEC O103:H25 NOS culture supernatants was digested with *Eco*RI and cloned in pUC18, and the clones were subsequently sequenced.

### GenBank accession numbers

The genome sequence of EHEC O103:H25 NOS has been deposited at DDBJ/EMBL/GenBank under the accession no AGSG00000000. The version described in this paper is the first version, AGSG01000000. The 61 kb Stx2 phage genome has accession no JQ011318 and the 24 kb early region of the *stx*-negative phage has accession no JQ011316. The 45 kb phi-like phage has accession no JQ011317.
